# Advance care planning conversations in primary care: a quality improvement project using the Serious Illness Care Program

**DOI:** 10.1186/s12904-021-00817-z

**Published:** 2021-07-30

**Authors:** Abe Hafid, Michelle Howard, Dale Guenter, Dawn Elston, Shireen Fikree, Erin Gallagher, Samantha Winemaker, Heather Waters

**Affiliations:** 1 Department of Family Medicine, McMaster University, Hamilton, Canada; 2 Department of Health Research Methods, Evidence & Impact, McMaster University, Hamilton, Canada; 3 Department of Family Medicine, Division of Palliative Care, McMaster University, Hamilton, Canada

**Keywords:** Family Practice, Advance Care Planning, Serious Illness Care Program, Quality Improvement

## Abstract

**Background:**

Advance care planning (ACP) conversations are associated with improved end-of-life healthcare outcomes and patients want to engage in ACP with their healthcare providers. Despite this, ACP conversations rarely occur in primary care settings. The objective of this study was to implement ACP through adapted Serious Illness Care Program (SICP) training sessions, and to understand primary care provider (PCP) perceptions of implementing ACP into practice.

**Methods:**

We conducted a quality improvement project guided by the Normalization Process Theory (NPT), in an *interprofessional academic family medicine group in Hamilton, Ontario, Canada*. NPT is an explanatory model that delineates the processes by which organizations implement and integrate new work. *PCPs (physicians, family medicine residents, and allied health care providers), completed pre- and post-SICP self-assessments evaluating training effectiveness, a survey evaluating program implementability and sustainability, and semi-structured qualitative interviews to elaborate on barriers, facilitators, and suggestions for successful implementation. Descriptive statistics and pre-post differences (Wilcoxon Sign-Rank test) were used to analyze surveys and thematic analysis was used to analyze qualitative interviews.*

**Results:**

30 PCPs participated in SICP training and completed self-assessments, 14 completed NoMAD surveys, and 7 were interviewed. There were reported improvements in ACP confidence and skills. NoMAD surveys reported mixed opinions towards ACP implementation, specifically concerning colleagues’ abilities to conduct ACP and patients’ abilities to participate in ACP. Physicians discussed busy clinical schedules, lack of patient preparedness, and continued discomfort or lack of confidence in having ACP conversations. Allied health professionals discussed difficulty sharing patient prognosis and identification of appropriate patients as barriers.

**Conclusions:**

Training in ACP conversations improved PCPs’ individual perceived abilities, but discomfort and other barriers were identified. Future iterations will require a more systematic process to support the implementation of ACP into regular practice, in addition to addressing knowledge and skill gaps.

**Supplementary Information:**

The online version contains supplementary material available at 10.1186/s12904-021-00817-z.

## Background

Advance care planning (ACP) is a discussion of: care options; patient beliefs, values and preferences; mental and physical prognoses; and care decisions not restricted to goals of care and resuscitation directives [1, 2]. ACP can occur over multiple interactions with healthcare providers as patient beliefs, values and preferences may change with time [3, 4]. Physician-led discussions of ACP with older adults (> 65 years old) are associated with increased quality of life and mood, longer survival, decreased use of non-beneficial medical care near death, decreased expenditures, enhanced goal-consistent care, and positive family outcomes [5, 6].

Canadians want more information about ACP from their healthcare providers [7]. A national survey of older patients from Canadian family practices reported that 68% of older patients have thought about their medical care preferences if they were sick and in hospital, but only 9% of them had engaged in discussion with their primary care physicians about their end-of-life care preferences [8]. Despite patients wanting to engage in ACP, such conversations are infrequently had or recognized in primary care settings. A structured literature review determined that the prevalence of general practitioner-led ACP conversations with older adults is approximately 21% globally [1].

Primary care providers have mixed perceptions of ACP and experience challenges in implementing this activity into practice. A national survey of Canadian primary care providers (PCPs) reported that PCPs are very confident and willing to have ACP conversations with their patients, yet reported low participation rates [9]. The same survey also identified key barriers to successful ACP implementation in primary care such as insufficient time and busy clinical schedules, lack of ACP knowledge or training, and patient end-of-life care literacy [10]. Despite these mixed perceptions, primary care is an ideal healthcare setting to initiate ACP conversations. PCPs share longitudinal relationships with their patients, which may make ACP conversations possible before critical illness or hospitalization, and free to span multiple encounters.

The Serious Illness Care Program (SICP), developed at Ariadne Labs in Boston, Massachusetts, is a communication intervention developed to identify oncology patients with a high risk of death in the next year and to train oncologists in having ACP conversations using the structured Serious Illness Conversation Guide (SICG) [11]. The SICP has also been used in primary care settings with evidence of acceptance and benefit [12]. This training involved the use of standardized patients, observation and providing feedback to participants. Apart from quality improvement initiatives in two primary care clinics affiliated with the Brigham and Women’s Hospital in Boston [12]. SICP implementation has not been evaluated in primary care settings extensively. As a result, there is an impetus to evaluate SICP implementation in Canadian primary care settings.

ACP conversations can sometimes feel difficult for providers and patients, and as a result, communication training must include comfortable language to initiate ACP conversations with patients. However, addressing the need for serious illness communication training among PCPs is only one of the strategies that will be needed to foster the implementation of ACP in primary care settings. Successfully implementing ACP in any healthcare setting involves steps such as the identification and preparation of appropriate patients and ensuring time is made available during appointments, that roles are clear, and that clear documentation of conversations occurs. The Normalization Process Theory (NPT) is a theory of action that is used for understanding and evaluating how complex interventions are implemented and embedded in the everyday work of health care [13]. NPT can assist with explaining success or failure of implementation, and highlight the specific constructs that are impeding implementation [14]. The core constructs are coherence (work that defines and organizes the objects of a practice); cognitive participation (work that defines and organizes the enrolment of participants in a practice); collective action (work that defines and organizes the enacting of a practice); and reflexive monitoring (work that defines and organizes the knowledge upon which appraisal of a practice is founded) [13].

As part of a quality improvement initiative, our goal was to explore the perceptions of clinicians in an interprofessional academic family practice regarding implementing ACP into routine care through using an adapted SICP and SICG. guided by NPT.

## Methods

This was a quality improvement project informed by a mixed-methods design, where both quantitative and qualitative data was collected, and interpretation of the data from both methods was done concurrently. This project was conducted at the McMaster Family Health Team; an academic interprofessional family medicine group consisting of two clinics with 40 primary care physicians, diverse allied health professionals and approximately 80 family medicine residents. The clinics provide care to approximatey 40,000 patients in Hamilton, Ontario, Canada.

### Program implementation and evaluation

From March to May 2018, all PCPs (physicians, registered nursing staff, and social workers) at the McMaster Family Health team were invited to three training SICP sessions through clinic communication channels. Thirty-four PCPs, consisting of 13 physicians, 12 residents, 4 nurse practitioners, 3 registered nurses, and 2 social workers, attended the training sessions, of whom 76% were women. Participants attended a training session consisting of group-based discussions about the principles of serious illness communication, followed by simulated conversations with standardized patients using the SICG, and receipt of real-time observation and feedback from trainers. The event followed the same 2.5-h format and used the same materials as those employed in a study in a hospital in the same city, focusing on skills practice with cases acted by simulated patients [15]. The primary care cases were those adapted by the program SICP developers for primary care [12]. Trainers had attended training themselves previously, from the team who developed the SICP. Upon completion, participants were asked to complete pre-and post-training self-assessment surveys using Likert scale responses (1 = Not at all skilled, 5 = Extremely Skilled) to evaluate their perception of their skills (Additional File 1).

### Evaluating the implementability of ACP in primary care

Following the training sessions, PCPs were approached to use the SICG to conduct conversations with two to three patients aged 65 or older with any diagnosis of a chronic, progressive illness or frailty that is expected to decrease life expectancy. PCPs agreed to participate in research and refer consenting patients and their substitute decision-makers to complete surveys evaluating their conversation experience. Upon completion of conversations with three patients, PCPs were to complete surveys evaluating their conversation experiences.

Surveys and interviews were used to measure PCP perceptions of ACP and the SICP in primary care, as per the NPT framework. We chose NPT to guide the evaluation because it is a theory of implementation that focuses on people and their behaviours both individually and collectively as part of a social system, [14] and it can provide empiric evidence of where there are gaps in implementation and progress over time [16, 17]. The constructs of NPT align well to implementing the multi-step emotionally laden process of ACP in a complex interprofessional environment such as primary care. We felt that NPT, and specifically the measurement tool NoMAD, would be the most informative approach to evaluating implementation that would highlight specific areas for improvement and importantly, where additional attention would not be needed. NPT has been used successfully to plan, monitor and improve new interventions in primary care [16, 17]. The NoMAD survey is a customizable 23-question survey based on the NPT constructs, used for gauging implementation processes from the viewpoint of healthcare professionals directly implementing new interventions [18, 19]. The survey has been psychometrically validated and demonstrates good internal consistency *(Cronbach’s alpha* = *0.89),* and good face and construct validity [19]. The survey was used to evaluate participants’ perceptions of implementing ACP in their practice (Additional File 2). Surveys were administered three months following the final workshop to participating trained PCPs, to allow time for patient encounters to occur where an ACP conversation would be relevant. Surveys were administered in-person or by e-mail, with bi-weekly in-person or email reminders for survey completion.

A sub-group of participating PCPs were invited to participate in one-on-one qualitative interviews identify perceived barriers and facilitators to successful implementation, and suggestions to improve implementation. Interviewees were identified through convenience sampling, among participants who completed a NoMAD survey. With the knowledge that implementation had not launched after the training, the interview questions were focused on the NPT concepts of coherence and cognitive participation. We asked about perceptions of the SICG, how it was being used and if not used what were the barriers, and how patients could be best identified for initiating conversations. Interviews were conducted by a senior male primary care physician and a junior male research assistant. Interviews were conducted in-person or by telephone, 4 months following the final workshops. Interviews were audio recorded but were not transcribed. Recordings and notes were not anonymized in order to identify differences in experiences due to clinical role.

### Analyses

Shapiro–Wilk tests determined that the pre- and post-workshop self-assessment scores were not normally distributed. Wilcoxon Sign-Rank tests were conducted to examine pre-and post-workshop self-assessment scores to assess changes in conducting ACP due to the training workshops. Means, frequencies, and proportions were calculated to describe NoMAD survey responses to describe participant perceptions of implementing ACP into primary care using the SICP and SICG. Statistical significance was assessed by a two-tailed p-value of < 0.05. Analyses were completed using IBM SPSS Statistics for Windows, Version 26.0.

Interview recordings and notes were independently analyzed by two junior research assistants to identify barriers, facilitators, and suggestions for successful implementation. The first male analyst was involved in the interview process, while the second female analyst was given orientation to the data and asked to independently conduct thematic analysis. Identified themes were classified to NPT constructs and components. Neither analyst was a healthcare professional, and both have been involved in research on the topic previously. Due to the brevity and focused nature of the interviews to elaborate on processes of implementing ACP, interviews were not transcribed, and coding of text was not undertaken. Thematic analysis was undertaken, using the interview questions as a framework. After independently identifying themes, the researchers met to discuss their analysis together to reach consensus on final themes and their alignment to NPT constructs, with a senior female health researcher reviewing final findings. The interviews augmented the results of the NoMAD survey by providing more specific insights into the next steps of changes in the clinic to remove barriers to having ACP conversations.

The Hamilton Integrated Research Ethics Board granted an ethics exemption to evaluate this training on the basis of quality improvement, waiving the need to consent clinician participants and allied health professionals. The research ethics board required that we obtain informed consent from participating patients and substitute decision makers.

## Results

### Training evaluations

Thirty out of 34 trained PCPs completed a pre-and post-training self-assessment evaluating their perception of the impact of the training session on their confidence and abilities to conduct ACP. Eighty-nine percent reported the training to be effective in improving their skills in conducting serious illness conversations, and 96% recommended the training to other healthcare providers. PCPs reported improvements in every assessment category following the training session and all pre- and post- training assessment differences were statistically significant (Table 1). The largest improvements were reported in asking patients about their sources of strength, inquiring about views on critical abilities, exploring views on trade-offs, and overall confidence in having serious illness conversations.

**Table 1:** Participant’s self-assessment of skill pre-and post-Serious Illness Conversation training (n = 30)

**Self-assessment category**	**Pre-Workshop Score**	**Post-Workshop Score**	**P-Value** ^a^
	**Median (IQR)**	**Median (IQR)**	
1. Set up a serious illness conversation	3 (2,3)	4 (3, 4)	< .001
2. Assess patient understanding of their illness	3 (3, 4)	4 (3, 4.25)	< .001
3. Ask patients about their preferences for information about the future	3 (1, 3)	4 (3, 4)	< .001
4. Share prognosis	2 (1, 5)	3 (3, 4)	< .001
5. Acknowledge and respond to patient emotion	4 (3, 4)	4 (3, 5)	.014
6. Allow silence	3 (2, 4)	4 (3, 5)	.001
7. Explore goals for future care	3 (3, 4)	4 (3, 4.25)	< .001
8. Inquire about fears and worries	3 (2, 4)	4 (3, 4.25)	< .001
9. Ask about sources of strength	2 (2, 3.25)	4 (3, 4)	< .001
10. Explore views on trade-offs	2 (2, 3)	3.50 (3, 4)	< .001
11. Inquire about views on critical abilities^b^	2 (2, 3)	4 (3, 4)	< .001
12. Explore views on family involvement^b^	3 (3, 4)	4 (3, 4)	< .001
13. Speak < 50% of time	3 (2, 4)	4 (3, 5)	.001
14. Overall confidence in having serious illness conversations	2 (2, 3)	4 (3, 4)	< .001

Notes: Likert Scale Responses: 1 = Not at all skilled, 5 = Extremely Skilled; ^a^ Wilcoxon Sign-Rank Test, α = 0.05; ^b^ *N* = 29 due to incomplete assessment

Twenty-five trained PCPs agreed to use the adapted SICG to conduct ACP conversations with their patients and subsequently refer them to study researchers, however, 4 PCPs withdrew from the project and only 3 patients were referred to researchers by December 2018. As a result, patient ACP conversation experiences were not evaluated.

### NoMAD survey and interview findings

Fourteen PCPs completed surveys evaluating their perceptions of implementing ACP into practice. Seventy-one percent of respondents were female, 50% were physicians and 50% had more than 6 years of clinical experience. When asked to rate how familiar they felt about conducting ACP in primary care, respondents reported a mean score of 6.8 (0 = Feels very new, 10 = Feels completely familiar). Respondents reported mean scores of 5.9 and 8.0 (0 = Not at all, 10 = Completely) when asked if ACP is currently part of their work and if they feel that ACP can become part of their work.

Respondents reported mixed opinions towards ACP in primary care (Table 2). All participants agreed on the potential value of conducting ACP in their professional role and the belief that participating in ACP is a legitimate part of the PCP role. All respondents agreed or strongly agreed that they are open to working with colleagues in new ways to make ACP possible in primary care and will continue to support ACP implementation in primary care. However, 58% did not agree that they can easily make ACP part of their clinical routine. Fifty-four percent lacked confidence in their colleagues’ abilities in conducting ACP, and 57% lacked confidence in their patients’ abilities to engage with them when discussing ACP. While 86% of respondents believed that enough training can be provided to staff to implement ACP, 50% believed that available resources are not adequate to support implementation.

**Table 2:** Responses to the survey on implementation of Advance Care Planning (ACP) in primary care (n = 14)

Statement based on NoMAD survey adaptation	Agree or Strongly Agree,
	**N (%)**
1. I can see how ACP differs from previous management of patients	10 (71.4%)
2. Staff in my organisation share understanding of the purpose of conducting ACP in primary care	7 (50.0%)
3. I understand how conducting ACP affects (changes) the nature or way I work	13 (92.8%)
4. I can see the potential value of conducting ACP in my professional role	14 (100.0%)
5. There are key people driving ACP in primary care settings	9 (64.3%)
6. I believe that participating in ACP is a legitimate part of my role	14 (100.0%)
7. I am open to working with colleagues in a new way to make ACP work in primary care settings	14 (100.0%)
8. I will continue to support the implementation of ACP in primary care	14 (100.0%)
9. I can easily make ACP (identify, invite, discuss & discuss) part of my daily work	6 (42.9%)
10. Conducting ACP can be disruptive to my previous working relationships	2 (14.3%)
11. I have confidence in my colleagues’ ability to conduct ACP^a^	5 (35.7%)
12. I have confidence in patients’ ability to engage in ACP discussions with me	6 (42.9%)
13. ACP can be carried out by people with appropriate skills	14 (100.0%)
14. Sufficient training can be provided to staff to implement ACP in primary care	12 (85.7%)
15. Sufficient resources are available to support ACP implementation in primary care	5 (35.7%)
16. I have received feedback about the effects of conducting ACP in primary care settings	2 (14.3%)
17. The staff I work with agree that conducting ACP in primary care settings is worthwhile	12 (85.7%)
18. I value the effects that incorporating ACP has had on my daily work	12 (85.7%)
19. I think feedback about conducting ACP could be used to improve it in the future	12 (85.7%)
20. I can modify how I conduct ACP in primary care	11 (78.6%)

^a^ *N* = 13 due to incomplete survey

All 25 participating PCPs were invited for interviews, but only 7 PCPs (3 physicians and 4 allied health professionals) agreed to participate. Physicians identified busy clinical schedules as a barrier, noting difficulty in having opportunistic ACP conversations with their patients. Other barriers identified by physicians included patient preparedness to engage in ACP conversations, and discomfort or lack of confidence in having ACP conversations. Allied health professionals (i.e., nurse practitioners, registered nurses, and social workers) identified discussing prognosis with patients as a barrier, emphasizing that prognostication is outside their scope of practice. Another barrier identified by allied health professionals included identifying appropriate patients for ACP conversations. Physician identified barriers resonated with the cognitive participation construct, while allied health identified barriers resonated with the coherence construct.

Interviewees did not identify facilitators to successful program implemented, and instead, identified the following suggestions to increase the quantity and quality of ACP conversations in primary care: conducting conversations over multiple appointments, increased collaboration between physicians and allied health professionals, providing patients ACP resources to improve engagement, and more training to normalize conversations as a standard of care for health care providers regardless of clinical role or specialty. Table 3 presents the identified barriers and suggestions to successful implementation according to the constructs of NPT, along with illustrative quotes.

**Table 3:** Barriers and suggestions for successful implementation of advance care planning (ACP) into primary care and their alignment to Normalization Process Theory (NPT) constructs, identified by interviewed primary care providers (n = 7)

Core Idea	Theme (Frequency)	Quotes	NPT Construct (Component)
Barriers	Physician busy clinical schedules serve as a logistical challenge for conducting ACP (4).	*“I think that intentionally setting aside time [for ACP] is so important, because [ACP] cannot ever happen opportunistically.” (Physician 1)* *“Biggest barrier that I could see is physicians saying I don’t have time, and how can we make them realize that you do?” (Registered Nurse 1)*	Cognitive Participation (Enrolment)
	Physician perceptions of patient preparedness for ACP (2).	*“The patient was taken aback, or very surprised, that, out of the blue at this visit, that you would bring in the topic of ACP.” (Physician 2)*	Cognitive Participation (Initiation)
	Clinician discomfort or lack of confidence in having ACP conversations (1).	*“There is still clinician discomfort around this whole topic, and so it is easier to say, “Oh I think this is as good as it gets” and not offer the follow-up because it is scarier and you’re really getting into [ACP] and you worry a little bit about what the patient’s response is going to be.” (Physician 2)*	Cognitive Participation (Legitimation)
	Allied health scope of practice limitations, namely with prognostication (4).	*“Unless I know that the patient has already discussed prognosis with the physician, then I feel that it is out of my scope to initiate [prognostication].” (Social Worker)* *“[Prognostication] is not something that registered nurses learn in school, we are told that prognostication is beyond the scope.” (Registered Nurse 1)*	Coherence (Individual Specification)
	Allied health difficulty in identifying appropriate patients for ACP conversations (2).	*“Identifying folks, because I see folks from 14 to 87-year-olds, so it is a wide scope. Unless someone is facing something life changing, I am not going to have this conversation with a 14-year-old, or a 37-year-old, because we are usually focused on something else, they came in for.“ (Social Worker)*	Coherence (Individual Specification)
Suggestions	Conducting ACP conversations over multiple visits (4).	*“I almost feel like, that we need to figure out a quick intro [to ACP] … and [invite them] back for another visit… because then, in my mind, it is more regimented and scheduled, and will prepare myself and the person coming in.” (Physician 2)*	Cognitive Participation (Enrollment)
	Increased collaboration between physicians and allied health for ACP (2).	*“I wonder, it might be something, they have already met with the doctor and have that conversation about prognosis, and then they say, ‘Maybe we can refer you to our social worker who has training in this’”. (Social Worker)* *“So, a practice team needs to appreciate that this is everybody’s responsibility, everybody’s role. It doesn’t just have to be [the doctors]. So how can we do this as a team, to make this work.” (Registered Nurse 1)*	Coherence (Communal Specification)
	Training to normalize ACP conversations, regardless of specialty or role, to reduce discomfort (2).	*“I actually think [ACP training] should not be limited to primary care, so I think it should be something taught in medical school. I think every specialty, maybe not exactly this exact discussion, but needs to be able to set up the conversation, assess peoples understanding, talk about prognosis, because whatever specialty you’re in, you will be talking about prognosis about some type of illness.”* (Physician 3) *“If you’re not delivering anything new, then you are not outside your scope of practice to talk about things, like decline, change, and illness trajectory. That is very much within the skilled RN scope of practice…for me that is very comfortable. For allied health that may not have had all the extensive training that I have had, and I can see why they would say that’s not for me. And why they would think that’s not within their scope. So, they would need more training to do it” (Registered Nurse 1)* *“We have to make sure that health care providers are comfortable talking about death and dying, and they’re not. North Americans aren’t comfortable talking about dying and death so how do we change that?” (Registered Nurse 1)*	Cognitive Participation (Legitimation)
	Providing patients ACP resources to improve engagement (2).	*“I usually direct [patients] to a website that has sheets for them to work on with their family member or friend, ‘Speak Up!’ they have a great worksheet, so I start the conversation, get the ball rolling, and then direct them to that resource. … And that’s the biggest thing, people love being able to have something after that conversation, or have some tools to work with, as opposed to having to remember everything we talked about”* (Social Worker)	Cognitive Participation (Activation)

## Discussion

### Key findings

In this quality improvement project where 34 PCPs were trained to have serious illness conversations through an adapted SICP and SICG, we examined training assessments and perceptions of the implementability of ACP in primary care. Overall, PCPs rated the SICP training workshops as highly effective in improving their skills and confidence in having ACP conversations in primary care. Our findings complement existing literature on PCP perceptions of SICP training workshops for primary care [12]. We also found high PCP confidence and willingness to conduct ACP, but low completion and/or reporting rates, which resonates with other research [20]. This may be attributed to logistical challenges raised by physicians such as busy clinical schedules, which may result in lower ACP conversation rates despite high PCP confidence. Our findings that busy clinical schedules are perceived to be a barrier for physicians complements existing literature on ACP implementation [20–22].

Our results identified differences between physicians and allied health professionals. Physicians reported high self-confidence in conducting ACP with patients themselves, but less confidence in their colleagues conducting ACP conversations. Allied health professionals reporting greater difficulty with discussing prognosis compared to physicians is also found in other studies [20]. This is further compounded by scope of practice limitations, another barrier specific to allied health professionals. Allied health professionals’ hesitancy to conduct ACP conversations due to limited scope of practice is a barrier identified in other studies as well [20].

Our results suggest that training alone will not lead to increased rates of ACP conversations in clinical practice, despite positive reception to the training and using broad patient eligibility criteria. It was interesting that perceptions that ACP could become part of routine work were higher than perceptions that it currently was implemented or perceptions of confidence in conducting ACP. Physician doubts about ACP implementation were largely explained as logistical challenges, and assumptions made about patient preparedness. Our results suggest difficulty in knowing how to arrange conversations with busy clinical workflows, and how to broach a sensitive subject effectively and comfortably. Despite the training being evidence-based and incorporating both simulated patients and feedback, the training may not be real-world case-based enough to have a positive impact on confidence and workflow challenges. Although the SICP recognizes the need for process changes in clinics such as mechanisms to identify patients, trigger the conversation and prepare patients, [11] it is important to confirm the relevance of these approaches locally in quality improvement initiatives.

### Limitations

Our study has four important limitations to consider. First, our findings are reflective of one academic family health team. As a result, our quantitative findings may not apply to other primary care teams and organizations, or other health care settings, from a statistical generalizability perspective due to the localized nature of this project and the small sample size. However, our qualitative findings may apply to other primary care providers from a naturalistic generalizable perspective, by resonating with their tacit clinical experiences. Second, only 14 out of 25 consenting PCPs completed NoMAD surveys, resulting in a 56% response rate. A higher response rate would have further strengthened the validity of our findings. Third, the NoMAD survey was not administered at baseline, and, therefore, the survey findings cannot be interpreted as evidence for the effectiveness of the SICP training but can be useful to consider where further efforts should be directed for implementation. Fourth, our objective was to evaluate whether there was a role for SICP in implementing ACP in primary care, and we did not undertake the study to increase the frequency of ACP conversations. However, a separate chart review of patients who died from January 2017 to December 2017 from the McMaster Family Health Team, found that 42% of decedents had a goals of care conversation, 32% discussed their illness understanding, 26% discussed patient values, beliefs and priorities going forward, and only 11% discussed prognosis [23]. A repeat chart review may be able to report on the potential impacts of SICP implementation on the frequency of ACP conversations. Despite these limitations, our findings complement existing literature and findings.

### Considerations for future implementation & research

Our findings of the barriers to implementing ACP resonate with the concept of a socioecological perspective of ACP [24] which considers issues at the level of the individual clinician, interpersonal relationships with patients, organizational and systems supports and cultural and legal adaptations.. From a systems perspective, PCPs may benefit from systematically identifying patients who could benefit from ACP, for example through electronic medical record queries of patients with life-limiting chronic illnesses, or by inviting all older patients for conversations regardless of their health status. Both approaches address concerns related to identifying appropriate patients for ACP. From the individual and interpersonal level perspectives, administrative support may facilitate conversations by incorporating ACP resources that engage patients directly, such as the Speak Up! workbook, [25] in the clinical process. This may alleviate PCP concerns surrounding patient preparedness to engage in ACP conversations at their scheduled appointments. PCPs may also benefit from training curated to address specific knowledge gaps identified to their clinical roles. Specifically, ACP training for allied health professionals should focus on discussing disease prognosis or natural history of progressive illnesses. Furthermore, future ACP implementations may benefit from a community-of-practice approach, that aims for gradual change over one to two years with ongoing knowledge sharing. Lastly, future research is needed to evaluate patient and substitute decision-maker experiences of ACP conversations and the effectiveness of conversations in primary care.

## Conclusions

Overall, our findings suggest physicians and allied health professionals working in primary care are very receptive to the idea of ACP but are less optimistic about the feasibility of implementing ACP into clinical practice. Attention to specific implementation concerns may help identify improvements for future iterations of ACP implementation.

## Supplementary Information

**Additional file 1.** Serious Illness Care ProgramTraining Workshop Assessment.

**Additional file 2.** Advance Care Planning NoMAD Survey.

## Data Availability

The datasets analyzed during the current study are available from the corresponding author upon reasonable request.

## Abbreviations

ACP: Advance care planning
SICP: Serious Illness Care Program
SICG: Serious Illness Conversation Guide
NPT: Normalization Process Theory
PCP: Primary care provider
