# Supplementary material for: Advance care planning conversations in primary care: a quality improvement project using the Serious Illness Care Program
Source: BMC Palliat Care. 2021 Jul 30;20:122. doi: 10.1186/s12904-021-00817-z (PMC8325252; doi:10.1186/s12904-021-00817-z)
Supplement: Supplementary file 1 — Additional file 1. Serious Illness Care ProgramTraining Workshop Assessment. [file 12904_2021_817_MOESM1_ESM.docx]

**Self-assessment of your change in skills**

We are interested in how you think your level of skill has changed in each of the following areas now that you completed this training, compared to before the training.

**1= Not at all skilled 2 =Somewhat skilled 3= Skilled 4= Very skilled 5= Extremely skilled**

| Your level of skill **BEFORE** | | | | | **Self-assessment categories** | Your level of skill **NOW** | | | | |
| --- | --- | --- | --- | --- | --- | --- | --- | --- | --- | --- |
| 1 | 2 | 3 | 4 | 5 | Set up a serious illness conversation | 1 | 2 | 3 | 4 | 5 |
| 1 | 2 | 3 | 4 | 5 | Assess patient understanding of their illness | 1 | 2 | 3 | 4 | 5 |
| 1 | 2 | 3 | 4 | 5 | Ask patients about their preferences for information about the future | 1 | 2 | 3 | 4 | 5 |
| 1 | 2 | 3 | 4 | 5 | Share prognosis | 1 | 2 | 3 | 4 | 5 |
| 1 | 2 | 3 | 4 | 5 | Acknowledge and respond to patient emotion | 1 | 2 | 3 | 4 | 5 |
| 1 | 2 | 3 | 4 | 5 | Allow silence | 1 | 2 | 3 | 4 | 5 |
| 1 | 2 | 3 | 4 | 5 | Explore goals for future care | 1 | 2 | 3 | 4 | 5 |
| 1 | 2 | 3 | 4 | 5 | Inquire about fears and worries | 1 | 2 | 3 | 4 | 5 |
| 1 | 2 | 3 | 4 | 5 | Ask about sources of strength | 1 | 2 | 3 | 4 | 5 |
| 1 | 2 | 3 | 4 | 5 | Explore views on trade-offs | 1 | 2 | 3 | 4 | 5 |
| 1 | 2 | 3 | 4 | 5 | Inquire about views on critical abilities | 1 | 2 | 3 | 4 | 5 |
| 1 | 2 | 3 | 4 | 5 | Explore views on family involvement | 1 | 2 | 3 | 4 | 5 |
| 1 | 2 | 3 | 4 | 5 | Speak <50% of time | 1 | 2 | 3 | 4 | 5 |
| 1 | 2 | 3 | 4 | 5 | Overall confidence having serious illness conversations | 1 | 2 | 3 | 4 | 5 |

1. Overall, how effective was this training in improving your skills in conducting serious illness conversations? (Please circle)

| Not at all effective | Somewhat effective | Neither effective nor ineffective | Mostly effective | Extremely effective |
| --- | --- | --- | --- | --- |

1. How likely are you to recommend this training to others? (Please circle)

| Not at all likely | Somewhat likely | Neither likely nor unlikely | Mostly likely | Extremely likely |
| --- | --- | --- | --- | --- |

1. What is the single most important thing you will take away from the training and apply in your work?

___________________________________________________________________________

___________________________________________________________________________

___________________________________________________________________________

___________________________________________________________________________

1. What information in this training surprised, inspired, and/or helped you most?

___________________________________________________________________________

___________________________________________________________________________

___________________________________________________________________________

___________________________________________________________________________

1. What could we do to improve this training?

___________________________________________________________________________

___________________________________________________________________________

___________________________________________________________________________

___________________________________________________________________________
