# Supplementary material for: Advance care planning conversations in primary care: a quality improvement project using the Serious Illness Care Program
Source: BMC Palliat Care. 2021 Jul 30;20:122. doi: 10.1186/s12904-021-00817-z (PMC8325252; doi:10.1186/s12904-021-00817-z)
Supplement: Supplementary file 2 — Additional file 2. Advance Care Planning NoMAD Survey. [file 12904_2021_817_MOESM2_ESM.docx]

**Part A: About yourself, the primary care clinician**

**Name:**

**Questionnaire Completion Date:**

1. **How many years have you worked as a primary care clinician?**

|  |  |  |  |  |  |
| --- | --- | --- | --- | --- | --- |
| Less than one year | 1 – 2 years | 3 – 5 years | 6 – 10 years | 11 – 15 years | More than 15 years |

1. **How would you describe your clinical role?**

Physician

Resident

Nurse Practitioner

Registered Nurse

Social Worker

Other, please specify:

**Part B: General questions about conducting advance care planning (ACP), identifying patients for ACP, and documenting ACP.**

| **When you conduct ACP in primary care, how familiar does it feel?** | | | | | | | | | | |
| --- | --- | --- | --- | --- | --- | --- | --- | --- | --- | --- |
| Still feels very new | |  |  |  | Somewhat |  |  |  | Feels completely familiar | |
|  | | | | | | | | | | |
| 0 | 1 | 2 | 3 | 4 | 5 | 6 | 7 | 8 | 9 | 10 |
|  |  |  |  |  |  |  |  |  |  |  |

**“**

| **Do you feel that ACP is currently a normal part of your work?** | | | | | | | | | | |
| --- | --- | --- | --- | --- | --- | --- | --- | --- | --- | --- |
| Not at all |  |  |  |  | Somewhat |  |  |  |  | Completely |
|  | | | | | | | | | | |
| 0 | 1 | 2 | 3 | 4 | 5 | 6 | 7 | 8 | 9 | 10 |
|  |  |  |  |  |  |  |  |  |  |  |

**“**

| **Do you feel that ACP, will become a normal part of your work?** | | | | | | | | | | |
| --- | --- | --- | --- | --- | --- | --- | --- | --- | --- | --- |
| Not at all |  |  |  |  | Somewhat |  |  |  |  | Completely |
|  | | | | | | | | | | |
| 0 | 1 | 2 | 3 | 4 | 5 | 6 | 7 | 8 | 9 | 10 |
|  |  |  |  |  |  |  |  |  |  |  |

**Part C: Detailed questions concerning ACP in primary care settings**

*For each statement, please select an answer that best suits your experience from Option A. If the statement is not applicable to you, please select an answer from Option B.*

|  | **Option A** | | | | |  | **Option B** | | |
| --- | --- | --- | --- | --- | --- | --- | --- | --- | --- |
| **Section C1 – What is…** | Strongly Agree | Agree | Neither agree nor disagree | Disagree | Strongly Disagree |  | Not relevant to my role | Not relevant at this stage | Not relevant to the intervention |
| 1. I can see how ACP differs from previous management of patients |  |  |  |  |  |  |  |  |  |
| 1. Staff in my organisation share understanding of the purpose of conducting ACP in primary care |  |  |  |  |  |  |  |  |  |
| 1. I understand how conducting ACP affects (changes) the nature or way I work |  |  |  |  |  |  |  |  |  |
| 1. I can see the potential value of conducting ACP in my professional role |  |  |  |  |  |  |  |  |  |

|  | **Option A** | | | | |  | **Option B** | | |
| --- | --- | --- | --- | --- | --- | --- | --- | --- | --- |
| **Section C2 – Who does it…** | Strongly Agree | Agree | Neither agree nor disagree | Disagree | Strongly Disagree |  | Not relevant to my role | Not relevant at this stage | Not relevant to the intervention |
| 1. There are key people driving ACP in primary care settings |  |  |  |  |  |  |  |  |  |
| 1. I believe that participating in ACP is a legitimate part of my role |  |  |  |  |  |  |  |  |  |
| 1. I am open to working with colleagues in a new way to make ACP work in primary care settings |  |  |  |  |  |  |  |  |  |
| 1. I will continue to support the implementation of ACP in primary care |  |  |  |  |  |  |  |  |  |

|  | **Option A** | | | | |  | **Option B** | | |
| --- | --- | --- | --- | --- | --- | --- | --- | --- | --- |
| **Section C3 – How does it get done?** | Strongly Agree | Agree | Neither agree nor disagree | Disagree | Strongly Disagree |  | Not relevant to my role | Not relevant at this stage | Not relevant to the intervention |
| 1. I can easily make ACP (identify, invite, discuss & document) part of my daily work |  |  |  |  |  |  |  |  |  |
| 1. Conducting ACP can be disruptive to my previous working relationships |  |  |  |  |  |  |  |  |  |
| 1. I have confidence in my colleagues’ ability to conduct ACP |  |  |  |  |  |  |  |  |  |
| 1. I have confidence in patients’ ability to engage in ACP discussions with me |  |  |  |  |  |  |  |  |  |
| 1. ACP can be carried out by people with appropriate skills |  |  |  |  |  |  |  |  |  |
| 1. Sufficient training can be provided to staff to implement ACP in primary care |  |  |  |  |  |  |  |  |  |
| 1. Sufficient resources are available to support ACP implementation in primary care |  |  |  |  |  |  |  |  |  |

|  | **Option A** | | | | |  | **Option B** | | |
| --- | --- | --- | --- | --- | --- | --- | --- | --- | --- |
| **Section C4 – Why did it happen like that?** | Strongly Agree | Agree | Neither agree nor disagree | Disagree | Strongly Disagree |  | Not relevant to my role | Not relevant at this stage | Not relevant to the intervention |
| 1. I have received feedback about the effects of conducting ACP in primary care settings |  |  |  |  |  |  |  |  |  |
| 1. The staff I work with agree that conducting ACP in primary care settings is worthwhile |  |  |  |  |  |  |  |  |  |
| 1. I value the effects that incorporating ACP has had on my daily work |  |  |  |  |  |  |  |  |  |
| 1. I think feedback about conducting ACP could be used to improve it in the future |  |  |  |  |  |  |  |  |  |
| 1. I can modify how I conduct ACP in primary care |  |  |  |  |  |  |  |  |  |

**SURVEY CONCLUSION** – Thank you for completing our survey!
